# The occurrence of dot-like micro B chromosomes in Korean field mice *Apodemus peninsulae* from the shore of the Teletskoye Lake (Altai Mountains)

**DOI:** 10.3897/CompCytogen.v14i1.47659

**Published:** 2020-03-04

**Authors:** Yuriy M. Borisov, Sergey A. Abramov, Marina Y. Borisova, Igor A. Zhigarev

**Affiliations:** 1 Severtsov Institute of Ecology and Evolution, Russian Academy of Sciences, Leninskii pr.33, Moscow 119071, Russia Severtsov Institute of Ecology and Evolution, Russian Academy of Sciences Moscow Russia; 2 Institute of Systematics and Ecology of Animals, SB RAS, ul. Frunze 11, Novosibirsk 630091, Russia Institute of Systematics and Ecology of Animals, Russian Academy of Sciences Novosibirsk Russia; 3 University of Nantes, UFR Medicine, 1 rue Gaston Veil, Nantes 44035, France University of Nantes Nantes France; 4 Moscow Pedagogical State University, Institute of Biology and Chemistry, ul. Kibalchicha 6, Moscow 129164, Russia Moscow Pedagogical State University Moscow Russia

**Keywords:** Apodemus (Alsomys) peninsulae, B chromosome dynamics, dot-like micro B chromosomes, karyotype

## Abstract

The data on the changes in the cytogenetic structure of the geographic population of Korean field mouse Apodemus (Alsomys) peninsulae Thomas, 1907 at the southern shore of the Teletskoye Lake (Altai Republic) are presented. In 1980 no dot-like microchromosomes were found in 34 mice captured on the southern and northern coasts of the Teletskoye Lake. In 2011, a 1.6-fold (from 2.7 to 4.3) increase in the mean number of B chromosomes compared to the rate estimated there earlier in 1980 was discovered. In 11 of the 15 mice (73%) captured in 2011, the karyotypes contained 1–2 dot-like micro B chromosomes and 1–5 macro B chromosomes. The pollution of the territory by the remains of the rocket fuel components may be an appropriate explanation for the cause of the karyological changes observed in *A. peninsulae* in this region.

## Introduction

The story of studying and describing B chromosomes (Bs) dates back to 1907, when Edmund Wilson (1907), working on hemipteran chromosomes, noticed those that appeared to be additional to the main karyotype and were present only in a fraction of individuals. However, the term ‘B chromosome’ was only established 11 years later. In 1928, Lowell Fitz Randolph working on variation in maize chromosomes proposed to call stable chromosomes of the standard complement ‘A chromosomes’, and those coming additional to the standard complement and being variable in number and morphology, ‘B chromosomes’ (Randolph 1928). B chromosomes were discovered very early in the history of cytogenetics. By 2004, Bs have been found in 55 of 4629 living species of mammals (Vujošević and Blagojević 2004).

Rubtsov and Borisov (2018) suggest several models of B chromosome origin, while the article by Vujošević et al. (2018), provides an updated list of 85 mammalian species with Bs, and gives a detail description of research experiments accomplished on these species. The research article by (Makunin et al. 2018) provides novel data on B chromosome content and evolution in the red fox (the first mammalian species with Bs, whose genome has recently been sequenced and assembled (Kukekova et al. 2018), and in the raccoon dog, the carnivore species, where B specific coding genes were discovered almost 13 years ago. Using new generation sequencing, the authors argue that the origin of B chromosome in these species is independent. Through the analysis of B content in different mammals they conclude a frequent and independent re-use of the same genomic regions in B chromosome formation. They suggest that such a re-use may be connected with gene functions. By definition, these chromosomes are not essential for the life of the species, their presence is not necessary for some (as a rule, for most) individuals of the species, thus, the population can consist of individuals with 0, 1, 2, 3 (etc.) В chromosomes, but limited to the population by their critical number, i.e., with the maximum known number of Bs in the individuals in the population (Vujošević and Blagojević 2004, Vujošević et al. 2018). B chromosomes have been reported in six species of the genus *Apodemus* (*A. peninsulae* Thomas, 1907, *A. agrarius* Pallas, 1771, *A. sylvaticus* Linnaeus, 1758, *A. flavicollis* Melchior, 1834, *A. mystacinus* Danford & Alston, 1877, *A. argenteus* Temminck, 1844). High frequencies of Bs were recorded particularly in *A. peninsulae* and *A. flavicollis* (Zima and Macholán 1995, Vujošević and Blagojević 2004, Borisov and Zhigarev 2018). Through the wide geographical range *A. peninsulae* karyotypes contain from 48 to 78 chromosomes and the vast majority of individuals of this species have supernumerary B chromosomes (Borisov and Zhigarev 2018).

Earlier (Borisov and Zhigarev 2018), we analyzed B chromosome variation in Korean field mouse *Apodemus peninsulae* (Rodentia, Muridae) based on a 40-year study of karyotypes collected from geographically distant populations in East Siberia, North Mongolia, China and the Russian Far East. In *A. peninsulae* up to five morphotypes were revealed. In the East Asian mouse, there are up to 30 B chromosomes differing in number and having a diverse morphology varying from dot-like micro pointed and small acrocentric B chromosomes to meta- and submetacentric B chromosomes of different size (small, medium and large) (Borisov and Zhigarev 2018). In Siberian mouse populations 1–10 macro B chromosomes, 1–30 dot-like micro B chromosomes and different combinations of macro and micro B chromosomes could be observed. While in mice populations of other regions individuals with no B chromosomes are frequent, all individuals in Siberian populations have B chromosomes of different types which make stable inheritable population systems (Borisov and Zhigarev 2018). Notably, it is customary for the Siberian geographical populations of the species to have a different number of B chromosome morphotypes, which together form stably heritable population systems.

The pattern of evolutionary dynamics of Bs can be distinctly different between geographical populations, and both the parasitic and the heterotic models can be applied to explain the maintenance of Bs in different populations. Further studies are desirable to improve our understanding of the complicated evolutionary dynamics of Bs in the *A. peninsulae* (Borisov 1990, 2008, Rubtzov et al. 2009, Rubtsov and Borisov 2018). The Korean field mouse has thus become a good mammalian model for studies of evolutionary dynamics and effects of Bs on the host genome (Rubtzov et al. 2009, Borisov and Zhigarev 2018, Rubtsov and Borisov 2018). B chromosome frequencies in *A. peninsulae* show temporal variation. Comparison of Bs from the population from Altai Republic, trapped in the 1980 and 2002, showed that a mean number of Bs in this population has almost tripled in 22 years (Rubtzov et al. 2009). The question is how widely this phenomenon occurred along the Teletskoye Lake shore. Earlier В. Kral (1971) and we in 1980 (Borisov 1990, 2008) studied the cytogenetic structure of the population of *A. peninsulae* on the southern shore of the Teletskoye Lake. Therefore, now we have an opportunity to compare these data with the data on the state of the population in 2011, i.e. 30 years later.

## Material and methods

**Figure 1.**
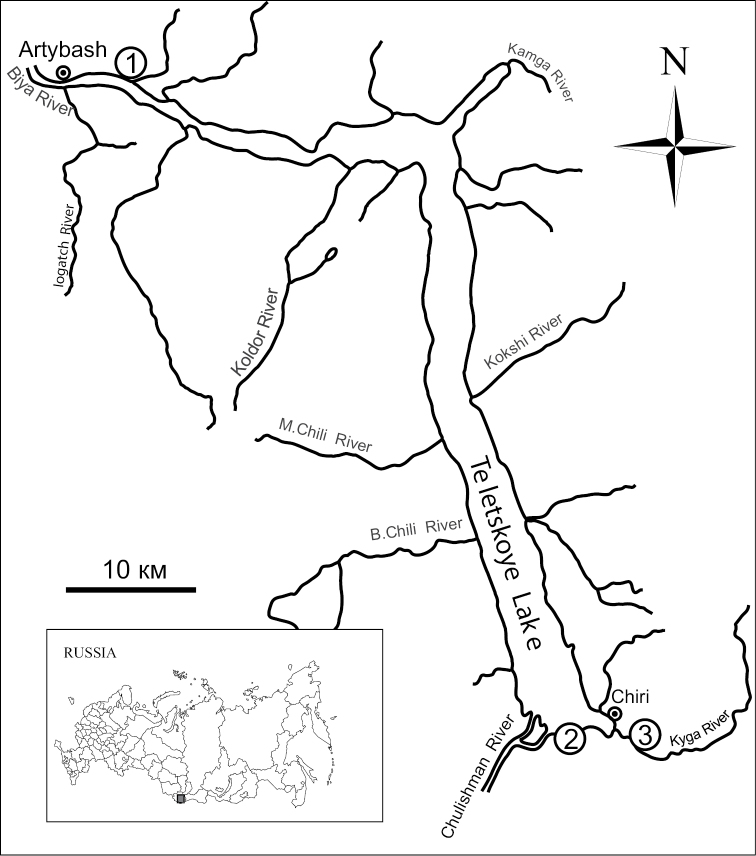
Schematic map of the study area and sampling localities of the mice *Apodemus peninsulae*: **1** locality in the vicinity of Artybash settlement **2** tourist centre at Karagai **3** locality at the Kyga River mouth.

We have analyzed new data on the chromosome sets of 15 *A. peninsulae* caught in 2011 in localities on the southern extremity of the Teletskoye Lake (Republic of Altai, Russia) (Fig. 1, localities Nos. 2 and 3): locality in the tourist centre at Karagai (4♀♀ and 7♂♂), locality at the Kyga River mouth in the vicinity of the settlement Chiri (The Altaiskiy State Nature Reserve) (3♀♀ and 1♂).

Chromosome preparations were made from marrow cells after intraperitoneal injection of 0.5 ml of 0.04% colchicine solution (Ford and Hamerton 1956). For defining the number and morphotypes of B chromosomes at least 20 metaphase cells from each animal were examined.

Previously, repeated DNA of Bs in the Korean field mouse has been analyzed by FISH with DNA probes generated by microdissection of A and B chromosomes followed by DOP-PCR (Karamysheva et al. 2002, Rubtsov et al. 2004). It was shown that all B chromosomes were composed of a large amount of repeated DNA sequences. The repeats were classified in terms of their homology and predominant location. But in this work, the routine dyeing was enough for the analysis of variability of B chromosome. B chromosomes were split by morphotype into five classes (Borisov and Zhigarev 2018), four of which are represented by macro B chromosomes more than 0.1 the size of the largest acrocentric chromosome (3.5–4.0 microns). The first class included large two-arm macro B chromosomes, from half the size of the largest autosome to the size comparable to that of the entire chromosome. The second class included smaller macro B chromosomes from 0.5 to 0.3 the size of the largest autosome. The third class was represented by small two-arm B chromosomes approximately 0.3–0.1 the size of the largest autosome. The fourth class comprised very rarely found and as a rule relatively small acrocentric B chromosomes, equal in sizes to B chromosomes of the third class. The fifth class included micro B chromosomes; these are dot-like В chromosomes with centromeres of unclear position. The frequency distribution of B chromosome classes was calculated from all metaphase plates of the studied individuals. Calculations of chromosome dimensions and registration of B chromosomes of *A. peninsulae* was performed using microscope D 5000 Leika.

## Results and discussion

**Figure 2.**
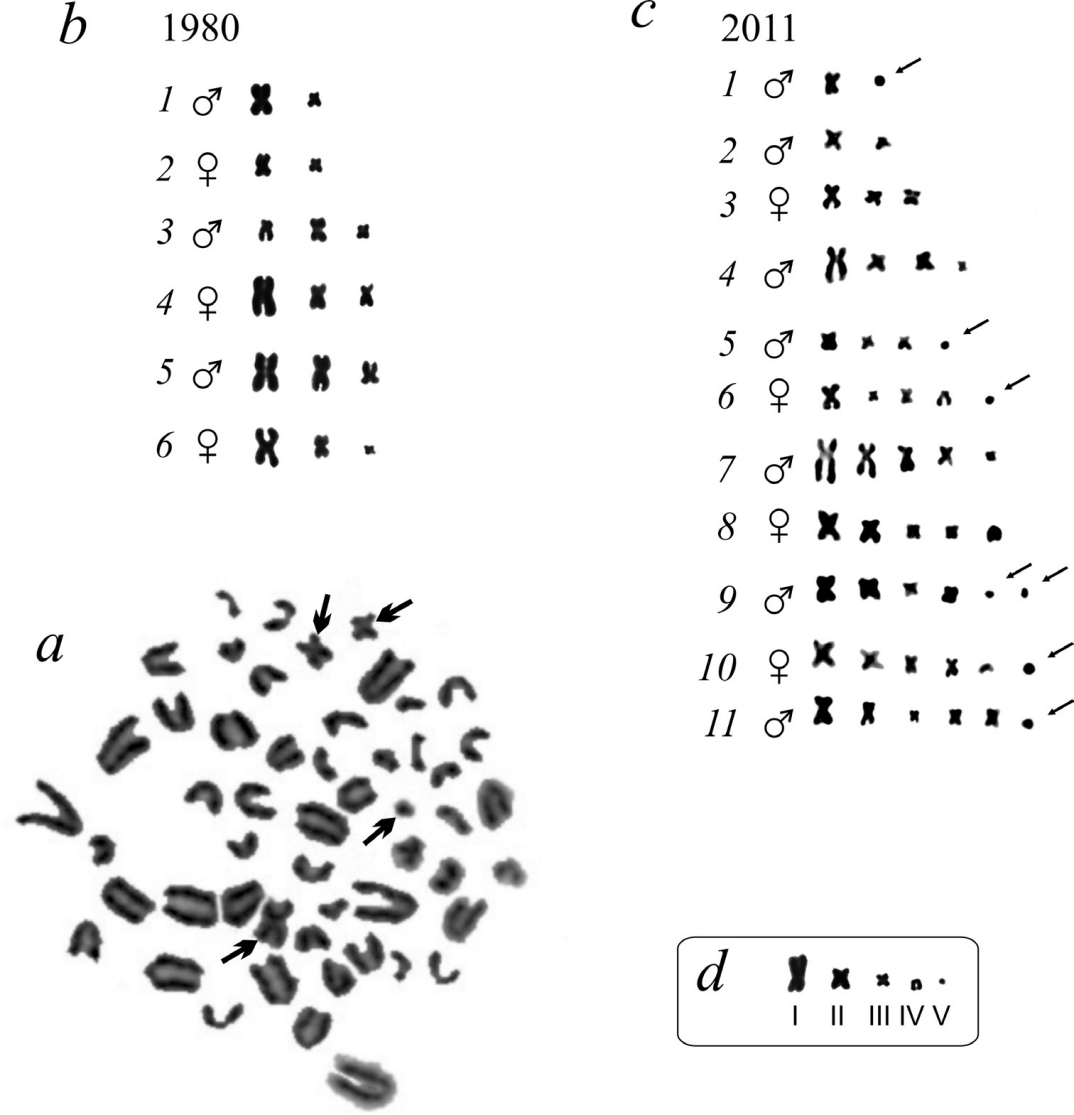
Metaphase plate of *Apodemus peninsulae* caught on the southern shore of the Teletskoye Lake in 2011 with different B chromosomes indicated with thick arrows (**a**) and the individual variants of the B chromosome system in mice from the southern shore of Teletskoye Lake revealed in 1980 (**b**) and 2011 (**c**) (arrows indicate the dot-like micro B chromosomes). Roman numerals denote five classes of identified B chromosomes (**d**).

The karyological analysis of 15 specimens of *A. peninsulae* from the southern shore of the Teletskoye Lake (Fig. 2, Table 1) captured in 2011 has shown that apart from the major stable chromosome set comprising 48 acrocentric A chromosomes, the karyotypes of these mice contained 2–6 B chromosomes. The variable part of the karyotype is represented by a combination of 0–1 large two-arm, 0–3 average two-arm, 0–3 small two-arm, 0–1 small acrocentric, and 0–2 dot-like micro B chromosomes. Small acrocentric and dot-like micro B chromosomes have been revealed in 14 (93%) of 15 studied mice from the southern coast of the Teletskoye Lake (Table 1).

**Table 1.** Locality, year of capture, and B chromosome system in *Apodemus peninsulae* from the southern shore of Teletskoye Lake.

No.	Sex	Variant of B chromosome system	Totall number of B chromosomes	B chromosome classes				
				I	II	III	IV	V
Karagai, 2011 (locality No. 2)								
1	♂	**x .**	2	0	1	0	0	1
2	♂	**x** ^	2	0	1	0	1	0
3	♀	**x x x**	3	0	2	1	0	0
4	♂	**x x x .**	3	1	2	0	0	1
5	♂	**x x x .**	4	0	0	3	0	1
6	♀	**x x x ^ .**	4	1	0	2	1	1
7	♂	**x x x x .**	5	1	1	2	0	1
8	♀	**x x x x** ^	5	1	1	2	1	0
9	♂	**x x x x . .**	5	0	2	2	0	2
10	♀	**x x x x ^ .**	6	1	1	2	1	1
11	♂	**x x x x x .**	6	1	3	1	0	1
**x̄_B_ (Karagai, 2011)**			**4.1**	**0.55**	**1.27**	**1.36**	**0.36**	**0.82**
Kyga River, 2011 (locality No. 3)								
12	♀	**x x x .**	4	0	1	2	0	1
13	♂	**x x x** ^	4	0	1	2	1	0
14	♀	**x x x x .**	5	1	1	2	0	1
15	♀	**x x x x .**	5	1	2	1	0	1
**x̄_B_ (Kyga River, 2011)**			**4.5**	**0.50**	**1.25**	**1.75**	**0.25**	**0.75**
**x̄_B_ (totall)**			**4.2** ± 0.33	**0.53** ± 0.13	**1.26** ± 0.21	**1.47** ± 0.24	**0.33** ± 0.13	**0.80** ± 0.15

**x̄_B_** – mean number of B chromosomes per individual (with standard error o mean).

This result differs from the result obtained from the same population in 1980 (Borisov 2008). We also observed an increase in the number of macro B chromosomes and occurrence of dot-like microchromosomes in karyotypes of the majority of specimens in *A. peninsulae* from the northern shore of the Teletskoye Lake in the vicinity of Artybash settlement (Fig. 1, locality No. 1) in 2006 as compared to 1980 (Fig. 2, d) (Borisov 1990, 2008). In 2011 the average number of B chromosomes in karyotypes of *A. peninsulae* increased 1.6-fold (from 2.7 to 4.2) (td ≈ 4.1, p >> 0.9999) (Table 1). Moreover, in 1980, in mice from the southern shore of the Teletskoye Lake no small acrocentric and dot-like microchromosomes were observed.

The study of 57 mice in 1971, 1978, 1980, 1986, 1988, and 1990 revealed no small acrocentric and dot-like microchromosomes (Kral 1971, Borisov 1990, 2008). For the first time small B chromosomes were discovered in four specimens of the Artybash population in 2002 (Borisov 2008). The subsequent study of the population in 2006 revealed an increase in the number of mice with small B chromosomes. Among 17 mice captured in 2006 small acrocentric and dot-like micro B chromosomes were revealed in 13 (76%) specimens (Borisov 2008). It has been established, therefore, that at present in populations of *A. peninsulae* inhabiting the shore of the Teletskoye Lake the process of reorganization of the B chromosome system is occurring. It consists in the growth of the average number of B chromosomes, due to, besides other factors, the increase in the number of small B chromosomes (Table 1, Fig. 2b).

There are not sufficient data for establishing the causes of the phenomenon discovered, so only suggestions with a certain degree of probability could be made. First of all, it is noteworthy that the changes in the B chromosome system of *A. peninsulae* have occurred throughout the past 30 years; therefore the factor responsible for them probably arose at the same period of time. According to the present concepts, the processes of the rise of dot-like micro B chromosomes of *A. peninsulae* and human small supernumerary marker chromosomes (sSMC) are associated with the increase in chromosome breaks on the boundaries of the pericentromeric heterochromatic regions. The reorganization of primary micro B chromosomes into macro B chromosomes is supposed to occur as a result of segment duplications and inversions of highly repetitive DNA sequences (Rubtzov et al. 2009, Rubtsov and Borisov 2018). The factors underlying the increase of chromosome ruptures resulting in formation of micro B chromosomes are possibly the impact of different mutagenic environmental factors or viral infections. No data on the prevalence of viral infections in *A. peninsulae* in the Altai Mountains are currently available. However, the Altai Mountains and Teletskoye Lake, in particular, are known as a region where space-rocket second stages containing remains of rocket propellant components, including one of the strongest mutagens – heptyl, have been falling for over 30 years (Panin and Perova 2006). Accumulation of heptyl in soil, vegetation cover and foodstuff of mice could be responsible for the karyological changes observed in *A. peninsulae* in that region. An indirect evidence that technogenic pollution destabilizes B chromosome systems of *A. peninsulae* is the fact that in another population of *A. peninsulae* inhabiting the territory extending for 200 km in the flood-lands of the Yenisei River left bank – the region under severe radiation pollution, karyotypes contained only dot-like micro B chromosomes (from 4 to 30) (Bolsunovsky et al. 2007).

The changes in the cytogenetic structure of the geographic mice population in the Altai Mountains (Borisov 2008, and the present report) and the microevolutionary processes occurring there are unique and require further study. The pollution of the territory by the remains of the rocket fuel components may be an appropriate explanation for the cause of the karyological changes observed in *A. peninsulae* in this region. The role of this and other new natural and man-caused factors affecting the nature of this region is yet to be studied. Possibility of an impact of migration on mice with such volatile karyotype is unlikely in our opinion.

All authors declare that there is no conflict of interests exists. All of the authors have contributed substantially to the manuscript and approved the submission.
